# Quantifying child growth effects using height-age instead of height-for-age z-scores in a meta-analysis of small-quantity lipid-based nutrient supplement trials

**DOI:** 10.1038/s41598-025-20664-9

**Published:** 2025-10-22

**Authors:** Kelly M. Watson, Alison S. B. Dasiewicz, Diego G. Bassani, Chun-Yuan Chen, Huma Qamar, Daniel E. Roth

**Affiliations:** 1 Centre for Global Child Health, The Hospital for Sick Children, 686 Bay St, Toronto, ON M5G 0A4 Canada; 2 Department of Nutritional Sciences, University of Toronto, Toronto, Canada; 3 Department of Paediatrics, University of Toronto, Toronto, Canada; 4 Dalla Lana School of Public Health, University of Toronto, Toronto, Canada

**Keywords:** Small-quantity lipid-based nutrient supplements, Child growth, Height-for-age z-scores, Length-for-age z-scores, Randomized controlled trials, Low- and middle-income countries, Height-age, Paediatric research, Randomized controlled trials, Nutrition

## Abstract

Height-age is the age at which growth-faltered children’s average observed height or length equals the median height or length of a child growth standard, corresponding to a length-for-age z-score (LAZ) of 0. In randomized controlled trials (RCTs) in low- and middle-income countries (LMICs), expression of linear growth outcomes using height-age may enhance the interpretability of intervention effects compared to conventional use of LAZ. Height-age can be used to derive the proportion of maximal benefit (PMB), whereby PMB = 0% indicates no effect and PMB = 100% indicates the intervention promoted growth at the rate expected for healthy children with the same starting height-age. In this proof-of-concept study, height-age and PMB were compared to LAZ in a meta-analysis of RCTs of small-quantity lipid-based nutrient supplements (SQ-LNS). Pooling across 15 trials in 10 LMICs, mean differences (MD; SQ-LNS minus control) in LAZ and height-age were 0.15 (95%CI: 0.12, 0.17) and 12 days (95%CI: 9, 14), respectively (N = 36,970). LAZ MD and height-age MD were highly correlated (rho = 0.74 overall and 0.94 upon exclusion of an outlier). The pooled PMB indicated that SQ-LNS achieves 11% of optimal growth potential (95% CI: [9.4, 12]; N = 19,768; 12 comparisons), but there was a substantial impact of between-trial heterogeneity (I^2^ = 90%). In conclusion, the effect of SQ-LNS on linear growth can be alternatively expressed in terms of height-age instead of LAZ. The PMB may enhance the interpretability of effect estimates by quantifying the extent to which an intervention improves growth in relation to a biological threshold, but further research is required to establish its validity and usefulness for assessing and comparing intervention effectiveness.

**Supplementary Information:**

The online version contains supplementary material available at 10.1038/s41598-025-20664-9.

## Introduction

Children’s average height has been widely used as an indicator of population health, nutrition, and overall well-being^1,2^. Length- or height-for-age z-scores (LAZ or HAZ; hereafter referred to as LAZ) are expressions of children’s observed growth relative to the World Health Organization (WHO) Growth Standards (WHO-GS) that define healthy growth for age and sex^3^. LAZ and stunting prevalence (% of children with LAZ <  − 2) are often specified as primary outcomes in randomized controlled trials (RCTs) assessing interventions aimed at improving child health and growth outcomes in low- and middle-income countries (LMICs).

The suitability of LAZ and stunting as RCT outcome measures has been questioned, as they are not expected to markedly change in response to single-component or short-term interventions^4,5^. For example, small-quantity lipid-based nutrient supplements (SQ-LNS) starting at about 6 months of age have shown promise in increasing mean LAZ and reducing stunting prevalence in LMICs^6,7^ but such findings have often been perceived as only modestly benefitting children’s linear growth^7–12^. Therefore, there is interest in alternative outcome measures that reflect the growth effects that could feasibly be achieved during a trial or programmatic intervention period^4^.

Height-age is the age at which a group of children’s observed average height (or length, if less than 2 years) would be considered normal according to a child growth standard (i.e., equal to the WHO-GS median, whereby LAZ = 0), and has been proposed as a candidate alternative to LAZ and stunting^13^. Height-age serves as a proxy for skeletal age, as the rate of long bone growth depends on the degree of growth plate maturation rather than chronological age; moreover, in a previously growth-faltered population, the complete removal of all growth constraints (i.e., inadequate dietary intake, infections, etc.) may be expected to lead to linear growth at approximately the rate expected for the height-age^14,15^. In previous work, we examined age-related trajectories of population-average linear growth using *growth delay* (chronological age minus height-age), which distinguished stable versus faltering growth patterns and led to different conclusions regarding the trajectory of population growth compared to those based on mean LAZ^16^. We also showed that growth delay, compared to LAZ, correlates more strongly and in the expected direction with other country-level indicators of health and well-being^17^. More recently, we developed methods to apply height-age in RCT analysis, and introduced a novel height-age-derived metric referred to as the ‘proportion of maximal benefit’ (PMB)^13^, which quantifies (as a %) the extent of children’s linear growth achieved by an intervention over a defined period relative to an optimal biological rate determined by the height-age at baseline.

The objective of this proof-of-concept study was to demonstrate the application of height-age and PMB in the context of a meta-analysis of RCTs of SQ-LNS, and to assess the extent to which the PMB may enhance the interpretability of estimated effects of an intervention on linear growth of children in LMICs.

## Methods

### Study design and data sources

This methodological study applies methods we recently developed for calculating height-age and PMB for trial analysis^13^. In a meta-analysis of SQ-LNS RCTs, we compared the inferences and interpretability of height-age and PMB estimates with LAZ, which is the conventional outcome used to quantify intervention effects on child linear growth. Height-age was derived from mean raw length (as reported directly, or back-calculated from reported mean LAZ) in published trials included in a recent systematic review and meta-analysis (SRMA) of the effect of SQ-LNS on child growth by Dewey et al. (2021)^7^. We closely followed the meta-analytical methods used in Dewey et al.’s SRMA to ensure consistency and enable a direct comparison between the re-expressed height-age results and conventionally reported results based on LAZ as per Dewey et al. Ethics board approval was not required for this secondary analysis of publicly available data and summary estimates from published trials.

### Study selection

Trials included in Dewey et al.^7^ were re-evaluated for inclusion in this study, and we also completed an updated literature search to identify any eligible trials published more recently and which were not included in Dewey et al.. Embase and Medline databases were searched from May 1, 2018 (1 year prior to the date of the search completed by Dewey et al. ^7^) to September 27th, 2022. An updated search using the same strategy was completed on August 16th, 2024 to check for any new studies published between 2022 to mid-2024. We adopted the search strategy used by Dewey et al. for these databases, which is detailed elsewhere^7,18^. The original database search in 2022 was supplemented by a manual search in Web of Science and Google Scholar for any studies that had cited Dewey et al.^7^. After removing duplicate titles, two co-authors independently screened titles and abstracts for full-text review using pre-defined eligibility criteria outlined below. Any disagreements about the studies to include were resolved through discussion.

### Eligibility criteria

The eligibility criteria outlined in Dewey et al.^7^, supplemented by two additional criteria specific to the present study, were used to screen studies. Inclusion criteria from Dewey et al.^7^ were: (i) studies conducted in LMICs, (ii) participants received SQ-LNS for at least 3 months during the 6–24 months age range, and (iii) the provided LNS was SQ (< ~ 125 kcal/d), or medium quantity-LNS (~ 250 kcal/d) if introduced at an appropriate age. In the absence of publicly-available IPD, additional inclusion criteria for this study to facilitate the calculation of height-age were: (i) study-level group mean length or LAZ reported, and (ii) WHO-GS was used to generate z-scores for trials that only reported LAZ.

Exclusion criteria in Dewey et al.^7^ and similarly applied here were: (i) studies in which severe or moderate malnutrition was an inclusion criterion, (ii) studies specifically done on diseased or hospitalized populations, (iii) studies that provided medium quantity-LNS or large quantity-LNS to children regardless of age range, (iv) studies for which the only available comparison was other types of supplementation (e.g., SQ-LNS versus fortified blended foods), and (v) studies which combined SQ-LNS with additional nutrition-specific intervention, and the comparator group did not allow the effect of SQ-LNS to be isolated.

### Data extraction

Two co-authors independently extracted data from published reports of the included studies. Where possible, preference was given to using individual participant data (IPD) available in public data repositories to generate relevant group-level summary estimates. Where IPD were not available, study-level estimates from the published reports were extracted from full-text publications. Authors of included studies were contacted via email to request further information or data as needed. If responses were not received after one week, authors were emailed again; if no response was received after two weeks, further attempts to contact authors ceased, but the study was still included if possible. Data extraction was conducted using Microsoft Forms (Supplementary Tables S1, S2). The conversion factor of 1 month = 30.4375 days was used to convert age units when necessary. Any discrepancies in extracted data were resolved through discussion, and involvement of additional team members if required.

For trials that included multiple relevant intervention and/or control groups, means and standard deviations of lengths and LAZs as well as sample sizes for each intervention group were aggregated using equations provided in the Cochrane handbook^19^ into one ‘SQ-LNS’ and one non-SQ-LNS ‘control’ group for each trial, using the groupings specified for the ‘all-trials analysis’ in Dewey et al.^7^. From the mean length estimates, height-age and PMB were subsequently calculated for each aggregated intervention and control group.

### Derivation of height-age from mean length

Height-age was determined by finding the age at which children’s observed average height (or length, if under 2 years) equals the median (i.e., LAZ = 0) of the WHO-GS^3^. Regardless of whether height or length was measured, all re-expressed linear growth outcomes are referred to as height-age (rather than length-age). Mean length was used directly to determine height-age if reported/available; otherwise, the reported mean LAZ and its upper and lower bounds of the 95% confidence interval (95% CI) were used to back-calculate the mean length (with 95% CI), from which height-age was then derived. Mean height-age was determined by finding the age corresponding to the WHO-GS median length^20^ which most closely reflected the reported or back-calculated mean length. A weighted average of the WHO-GS parameters (median length and coefficient of variation for age, which are disaggregated by sex) was used when the ratio of females to males was reported in the trial; otherwise, an equal ratio was assumed, and a simple average was taken. The same approach was used to convert the lower and upper bounds of the 95% CI for mean length to generate the corresponding bounds of the 95% CI for height-age. Methods for calculating height-age have been detailed elsewhere^13^, and code scripts for calculating height-age from mean LAZ^16^ and length^21^ are publicly available.

### Calculation of the proportion of maximal benefit (PMB)

Based on each group’s change in height-age during the intervention period (end-line height-age minus baseline height-age) (∆HA), PMB (expressed as a %) was calculated using the following formula^13^:

$$PMB=\frac{{\Delta HA}_{I}-{\Delta HA}_{C}}{{\Delta CA}_{I}-{\Delta HA}_{C}}\times 100$$

The subscript *I* represents the intervention (SQ-LNS) group, subscript *C* represents the control group, and ∆CA is the change in chronological age, the benchmark of optimal growth. The same formula was used to calculate the upper and lower bounds of the 95% CI using the upper and lower bounds of the 95% CI, respectively, for both ∆HA_*I*_ and ∆HA_*C*_^13^. Optimal growth occurs when height-age increases in parallel with chronological age (i.e., PMB = 100%) whereas maturation that is the same as in the control group corresponds to a PMB = 0%. It is possible for PMB to be < 0% if maturation is slower in the intervention versus the control group. Steps for calculating the PMB are outlined in Supplementary Figure S1, and additional explanation and rationale for this metric are provided elsewhere^13^. Height-age and PMB estimates for each trial were calculated using Stata version 17^22^.

### Statistical analyses

A dataset comprised of the mean raw length and LAZ at end-line, height-age MD at end-line, and PMB (where available) for each trial, with associated standard errors (SE), derived from 95% CIs, was compiled and used for subsequent meta-analysis in Stata version 17^22^.

Pooled measures of effect (LAZ MD, height-age MD, and PMB) were estimated with 95% CIs by combining trials using an inverse-variance (IV) fixed-effects model (to align with the primary approach used by Dewey et al.) and visualized using forest plots. The proportion of total variance attributable to between-study heterogeneity (*I*^2^) with 95%CIs was also calculated for each meta-analysis. Pairs of corresponding height-age MD and LAZ MD were plotted, and Spearman correlations coefficients were estimated and tested for significance at an alpha level of 5%.

Sensitivity analyses included: (1) random-effects instead of fixed-effects models; (2) restricted analysis including only those trials for which we generated effect estimates from IPD analyses; and (3) restricted analysis of trials of trials with available IPD with re-calculation of LAZ and height-age effect estimates accounting for clustering of outcomes due to the cluster-randomization trial design of these studies by calculating the intra-class correlation coefficient of endline LAZ and average cluster size to estimate the design effect and effective sample size^23^. We also performed a supplementary meta-analysis of the effect of SQ-LNS versus control on mean raw length at endline, using the same approach as the primary meta-analyses, for trials for which required data were available (Supplementary Table S3).

### Supplementary analysis: simulation

We performed a simulation study to assess the extent to which PMB is biased by baseline differences in length (and hence, height-age) between intervention and control groups. We simulated RCTs with 10,000 total participants conducted between 6 and 18 months of age with null treatment effects or positive treatment effects (corresponding to a 0.1 SD or 0.5 SD increase in LAZ at endline in the intervention compared to control group), and varying levels of baseline between-group differences in height-age. Parameters that were varied in this simulation were: (1) overall population mean LAZ (range: − 0.6 to − 2) to simulate varied levels of faltered populations; (2) correlation between baseline LAZ and an intervention group assignment variable (range: − 0.3 to 0.3) to simulate varying levels of baseline differences in height-age between the intervention group (n = 5000) and control group (n = 5000); and, (3) magnitude of the treatment effect (0, 0.1, or 0.5 absolute between-group differences on the LAZ scale at endline). The overall population mean LAZ was constant at baseline and endline and standard deviation was fixed at 1 for all simulation analyses, and we assumed normal distributions of LAZ at baseline and endline. The correlation between individual-level baseline and endline LAZ was set to 0.813, drawn from a published correlation matrix of mean LAZ for specified age intervals^24^. We calculated height-age at baseline and endline and PMB in each intervention and control group for every iteration of the simulation. PMB was visualized as a function of baseline differences in height-age using locally weighted scatterplot smoothing (LOWESS) curves, stratified by overall mean LAZ and magnitude of the treatment effect.

## Results

**Fig. 1:**
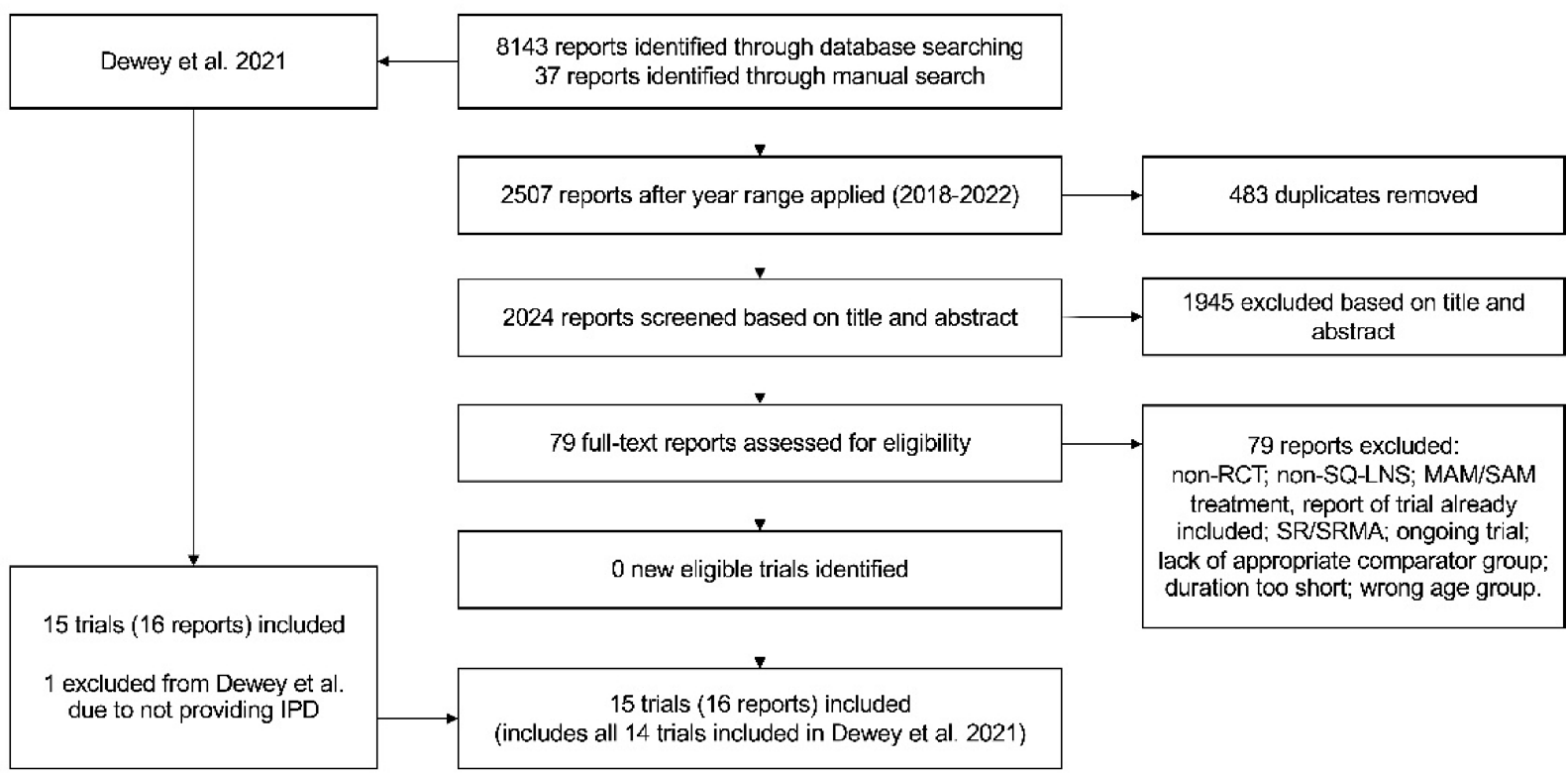
Study flow diagram. IPD, individual participant data; RCT, randomized controlled trial; SQ-LNS, small-quantity lipid-based nutrient supplements; MAM, moderate acute malnutrition; SAM, severe acute malnutrition.

In the initial literature search conducted in September 2022, 94 reports were selected for full-text review, of which 15 were eligible for inclusion, 14 of which had been included in Dewey et al. (Fig. 1). No additional trials were identified in the updated search in August 2024. Sample sizes ranged from 178 to 6583 children, average ages at the start of the infant intervention period ranged from 5.9 to 9.4 months, and the most common duration of SQ-LNS supplementation was 12 months (Range: 6–18) (Table 1). Among the trials in the SQ-LNS meta-analysis for which baseline length/LAZ data were available (n = 12 comparisons), between-group differences in baseline height-age ranged from 0 to 21 days; however, excluding the HAITI trial (in which the differences were related to a between-group discrepancy in chronological age at baseline), between-group differences in baseline height-age ranged from 0 to 5 days, and 7 of 11 trials had differences of 2 days or less. In the original published reports of the 15 included trials, 9 reported that SQ-LNS led to a significant improvement in linear growth, 2 did not report the effect on linear growth (PROMIS trials), and 5 reported no effect on linear growth (Table 1). In 4 of the 9 reports that reported SQ-LNS led to an improvement, the effect on linear growth was referred to as “modest” (Table 1).

**Table 1:** Characteristics of included trials.

Country; trial; design; duration (months); follow-up; first author year	Group (relevant trial arms combined), sample size at baseline (n)	Baseline age^1^ (SD), months	Summary of findings & interpretation as modest, if applicable
Bangladesh; JiVitA; cluster RCT; 12; longitudinal; Christian 2015	LNS (Plumpy’Doz + IYCF; chickpea-based LNS + IYCF; rice-lentil LNS + IYCF), n = 3160	6.2 (0.25)	Mean LAZ at 18 months was higher by 0.07–0.10 (all *P* < 0.05), in all treatment groups relative to the control. “Modestly increased linear growth…”
	Control (IYCF), n = 1438	6.1 (0.00)	
Bangladesh; RDNS; cluster RCT; 18; longitudinal; Dewey 2017^2^	LNS (maternal LNS & child LNS; maternal IFA & child LNS), n = 1651	6.1 (0.1)	Mean LAZ was 0.13 higher in LNS-LNS group compared to IFA-MNP at 24-months (*P* = 0.011). “Modest improvements in linear growth status among children provided with LNSs…”
	Control (maternal IFA), n = 793	6.1 (0.1)	
Bangladesh; WASH-Benefits; cluster RCT; 18; cross-sectional surveys; Luby 2018^3^	LNS (SQ-LNS + IYCF; WASH + SQ-LNS + IYCF), n = 1158	NA	Mean LAZ was 0.25 higher in SQ-LNS + IYCF group compared to passive control (95% CI: 0.15, 0.36). “Nutrient supplementation and counselling modestly improved linear growth…”
	Control (passive; WASH; handwashing; sanitation; water), n = 3426	NA	
Burkina Faso; iLiNS-ZINC; cluster RCT; 9; longitudinal; Hess 2015	LNS (LNS-Zn0; LNS-Zn5; LNS-Zn10; LNS-TabZn5), n = 2435	9.4 (0.35)	SQ-LNS provision, with or without zinc, increased mean LAZ by 0.27 at 18 months compared to control (*P* < 0.0001)
	Control (passive), n = 785	9.4 (0.35)	
Burkina Faso; PROMIS; cluster RCT; 12; longitudinal & cross-sectional; Becquey 2019^4,5,6^	LNS (SQ-LNS + IYCF), n = 916; 528	6.0 (0.4); NA	NA – did not report effect on linear growth
	Control (active), n = 964; 544	6.0 (0.4); NA	
Ghana; GHANA; RCT; 6; longitudinal; Adu-Afarwuah 2007^7^	LNS (SQ-LNS), n = 97	6 (NA)	Mean LAZ not significantly greater in SQ-LNS group compared to control
	Control (NI), n = 81	6 (NA)	
Ghana; iLiNS-DYAD-G; RCT; 12; longitudinal; Adu-Afarwuah 2016^8^	LNS (maternal & child LNS), n = 351	6 (NA)	Based on supplements received, SQ-LNS increased mean LAZ by 0.28 (95% CI: 0.10, 0.46) compared to IFA and was not statistically different from MMN at 18-months
	Control (maternal MMN; maternal IFA), n = 702	6 (NA)	
Haiti; HAITI; RCT; 6; longitudinal; Iannotti 2014	LNS (SQ-LNS for 6 months), n = 202	7.8 (1.7)	SQ-LNS for 6 months increased LAZ by 0.13 compared to control group, with adjustment for child age (*P* < 0.001)
	Control (active), n = 191	7.2 (1.7)	
Kenya; WASH-Benefits; cluster RCT; 18; cross-sectional surveys; Null 2018^3^	LNS (nutrition: SQ-LNS + IYCF; WASH + nutrition), n = 1455	NA	Mean LAZ was 0.13 higher in nutrition group compared to active control (95% CI: 0.01, 0.25). “LNS can modestly reduce growth faltering, but falls short of eliminating it…”
	Control (passive; active; WASH; handwashing; sanitation; water), n = 5128	NA	
Madagascar; MAHAY; cluster RCT; 6–12; longitudinal; Galasso 2019^9^	LNS (T2: child SQ-LNS + IYCF; T3: maternal & child SQ-LNS + IYCF), n = 1460	NA	None of the interventions (T1-T4) led to a significant improvement in LAZ compared to control (T0)
	Control (T0: control; T1: IYCF), n = 1455	NA	
Malawi; iLiNS-DYAD-M; RCT; 12; longitudinal; Ashorn 2015	LNS (maternal & child LNS), n = 192	6 (NA)	At 18 months, SQ-LNS did not increase mean LAZ compared to MMN and IFA
	Control (maternal MMN; maternal IFA), n = 433	6 (NA)	
Malawi; iLiNS-DOSE; RCT; 12; longitudinal; Maleta 2015	LNS (SQ-LNS w/ milk (10 g/d); SQ-LNS w/ milk (20 g/d); SQ-LNS w/o milk (20 g/d)), n = 966	5.9 (0.3)	No effect of SQ-LNS on linear growth from 6 to 18 months of age compared to control, regardless of whether SQ-LNS contained milk
	Control (active), n = 320	5.9 (0.3)	
Mali; PROMIS; cluster RCT; 18; longitudinal & cross-sectional; Huybregts 2019^4,5,6^	LNS (SQ-LNS + BCC^10^), n = 563; 923	6.5 (0.3); NA	NA – did not report effect on linear growth
	Control (IYCF + BCC^10^), n = 556; 947	6.5 (0.3); NA	
South Africa; TSWAKA; RCT; 6; longitudinal; Smuts 2019^11^	LNS (SQ-LNS; SQ-LNS-plus), n = 500	6.2 (0.25)	SQ-LNS formulations did not significantly improve linear growth compared to control at 12-months
	Control (NI), n = 250	6.2 (0.24)	
Zimbabwe; SHINE (HIV-); cluster RCT; 12; longitudinal; Humphrey 2019^12^	LNS (IYCF (includes SQ-LNS); IYCF + WASH), n = 1340	7.0 (1.65)	SQ-LNS intervention groups increased mean LAZ by 0.16 (95% CI 0.08–0.23) compared to active control and WASH groups
	Control (active; WASH), n = 1231	7.1 (1.7)	
Zimbabwe; SHINE (HIV +); cluster RCT; 12; longitudinal; Prendergast 2019^12^	LNS (IYCF (includes SQ-LNS); IYCF + WASH), n = 274	7.2 (1.4)	SQ-LNS intervention groups increased mean LAZ by 0.26 (95% CI 0.09–0.43) compared to active control and WASH groups. “…the absolute effect of the IYCF intervention on mean LAZ… was modest…”
	Control (active; WASH), n = 240	7.1 (1.44)	

AM, acute malnutrition; BCC, behaviour change communication; IFA, iron and folic acid supplementation; IYCF, infant and young child feeding; LAZ, length-for-age z-score; LNS, lipid-based nutrient supplements; MMN, multiple micronutrient; MNP, micronutrient powder; NI, no intervention; RCT, randomized controlled trial; RDNS, Rang Din Nutrition Study; SD, standard deviation; SQ, small-quantity; WASH, water, sanitation, and hygiene.

^1^ If the baseline age was not reported, average age was assumed to equal the baseline measurement timepoint.

^2^ Contacted authors of RDNS trial for mean age and SD, by group, at 6-months.

^3^ No appropriate baseline for this study due to cross-sectional follow-up; therefore, only end-line measurements included.

^4^ Did not report effect on linear growth, but individual participant data was publicly available and used to generate outcomes of interest for this study.

^5^ Cross-sectional and longitudinal cohorts were considered as separate comparisons; no appropriate baseline for cross-sectional cohorts.

^6^ N and age reported as “longitudinal; cross-sectional” for PROMIS trials.

^7^ In GHANA trial, baseline data was not collected for the control group; therefore, only end-line measurements were included.

^8^ Protocol violation in the iLiNS-DYAD-G trial, such that the intended treatment did not reflect actual treatment. Data were extracted based on supplements received.

^9^ No appropriate baseline, as several different age cohorts were eligible for SQ-LNS supplementation, and the intervention duration differed for each cohort.

^10^ Behaviour change communication (BCC) covered topics including nutrition, health, and hygiene.

^11^ Study not included in Dewey 2021 because individual participant data was not provided; however, individual participant data were not required for this meta-analysis.

^12^ Results of the SHINE trial were reported separately for children unexposed to HIV (mothers HIV-) and children exposed to HIV (mothers HIV+).

From among the 15 trials, 18 SQ-LNS-versus-control comparisons were included in the pooled analyses of LAZ MD and height-age MD. The SHINE trial in Zimbabwe contributed 2 comparisons because results were reported separately for children with mothers who tested positive for HIV (SHINE-HIV( +))^11^ and those who tested negative (SHINE-HIV( −))^25^. The PROMIS trials in Burkina Faso^26^ and Mali^27^ each included cross-sectional and longitudinal cohorts, so we adhered to the approach of Dewey et al. which included these as separate comparisons in their analyses^7^ (i.e., each trial contributed 2 comparisons). Publicly-available IPD were used for 6 comparisons from 4 trials (Supplementary Table S3). Due to missing baseline data for intervention and control groups for 6 comparisons, PMB was generated for only 12 comparisons (Supplementary Table S3). Mean length was reported and directly re-expressed as height-age for 9 trials (11 comparisons), mean LAZ was used to generate the mean length estimates for 4 trials (5 comparisons), and a combination of these two approaches (e.g., mean length at baseline and mean LAZ at end-line) was required for 2 trials (Supplementary Table S3).

**Fig. 2:**
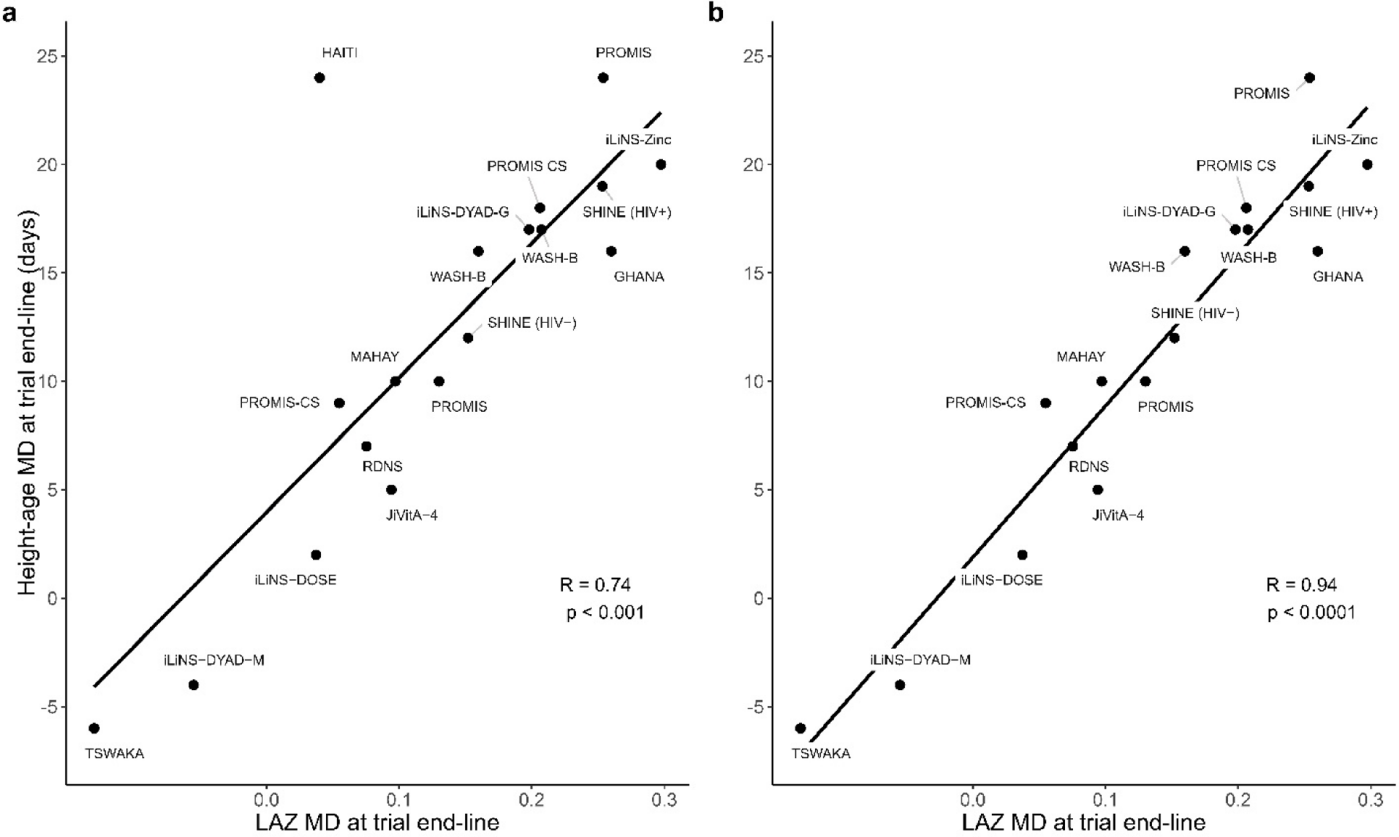
SQ-LNS effect expressed using height-age versus LAZ, including (Panel a) and excluding (Panel b) the ‘HAITI’ trial. Effect size for both height-age and LAZ is the mean difference (MD: intervention—control) at trial endline. Spearman correlation (R) reported, tested at a significance level of 5%. LAZ, length-for-age z-score; SQ-LNS, small-quantity lipid-based nutrient supplements.

**Fig. 3:**
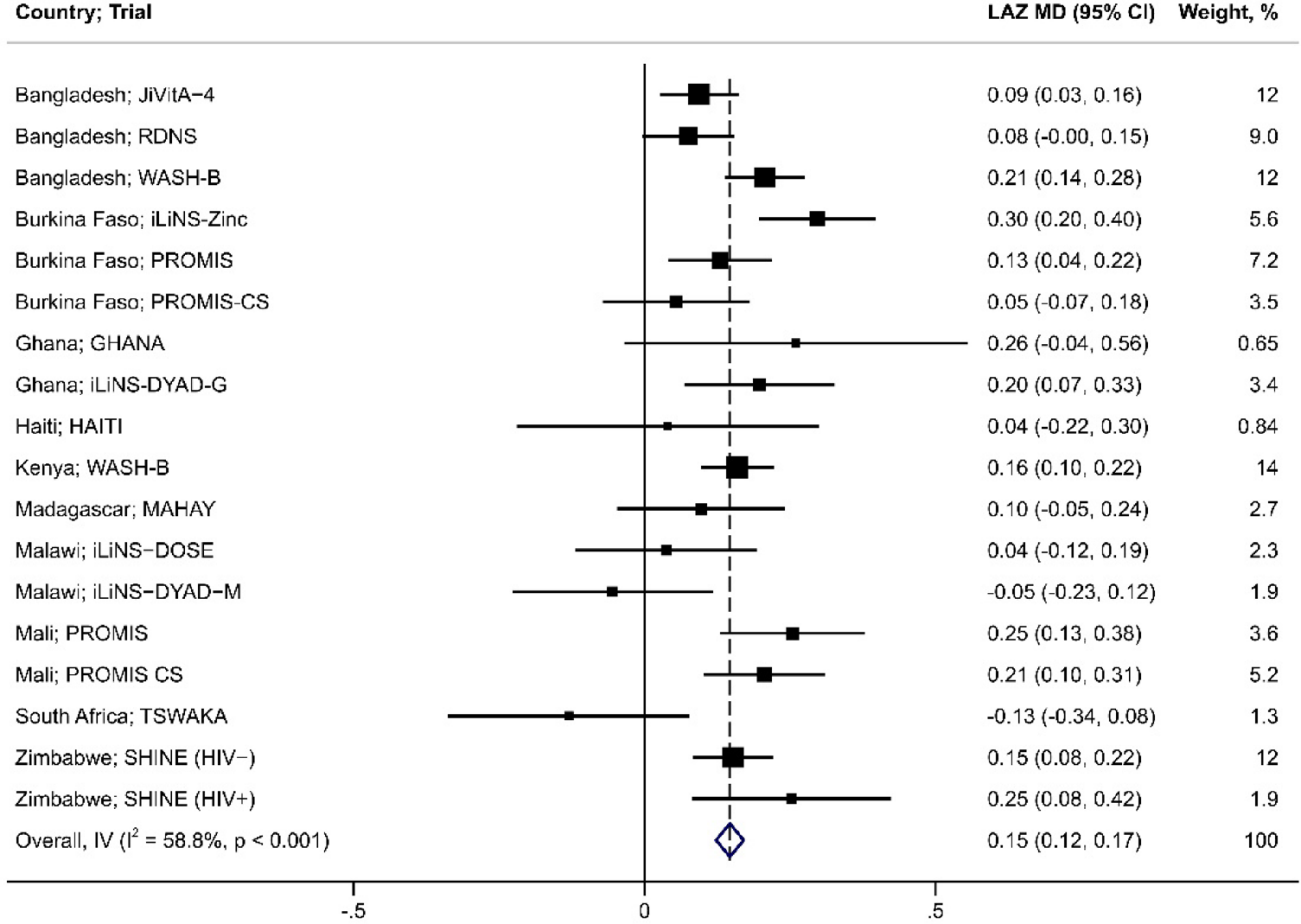
Effect of SQ-LNS expressed as the LAZ mean difference (SQ-LNS-control) at trial endline (n = 36,970 participants, 18 comparisons). The results of individual trials were pooled to estimate an overall effect size using an inverse variance (IV) fixed-effects model. Blue diamond is the pooled effect with 95% CI; black boxes are centered at the point estimates for each comparison and the size of the box corresponds to its relative weight; horizontal lines extend to the lower and upper bounds of the 95% CI for the estimate for each comparison. CI, confidence interval; LAZ, length-for-age z-score; MD, mean difference; SQ-LNS, small-quantity lipid-based nutrient supplements.

**Fig. 4:**
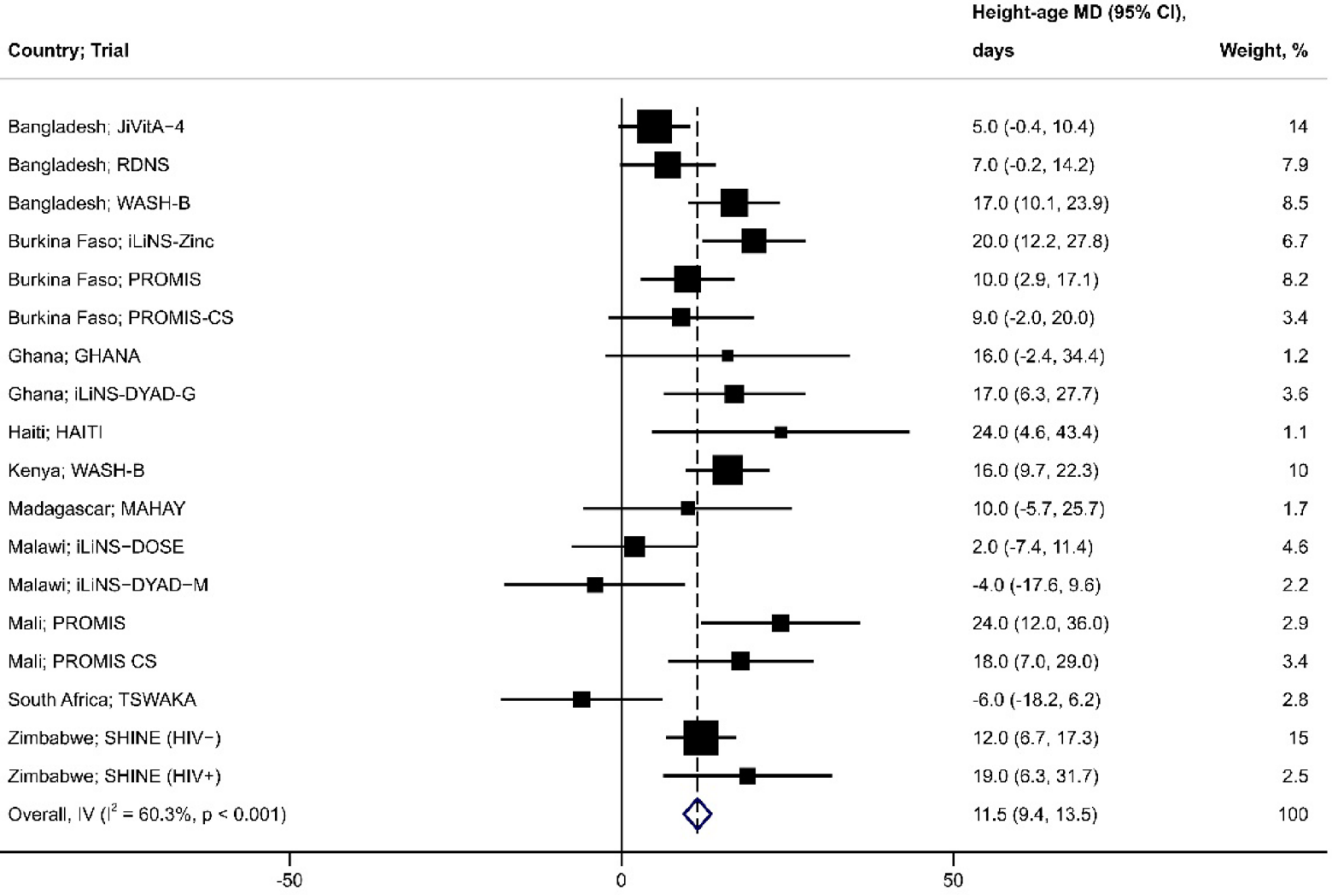
Effect of SQ-LNS expressed as the height-age mean difference (SQ-LNS-control) at trial endline (n = 36,970 participants, 18 comparisons). The results of individual trials were pooled to estimate an overall effect size using an inverse variance (IV) fixed-effects model. Blue diamond is the pooled effect with 95% CI; black boxes are centered at the point estimates for each comparison and the size of the box corresponds to its relative weight; horizontal lines extend to the lower and upper bounds of the 95% CI for the estimate for each comparison. CI, confidence interval; HA, height-age; MD, mean difference; SQ-LNS, small-quantity lipid-based nutrient supplements.

**Fig. 5:**
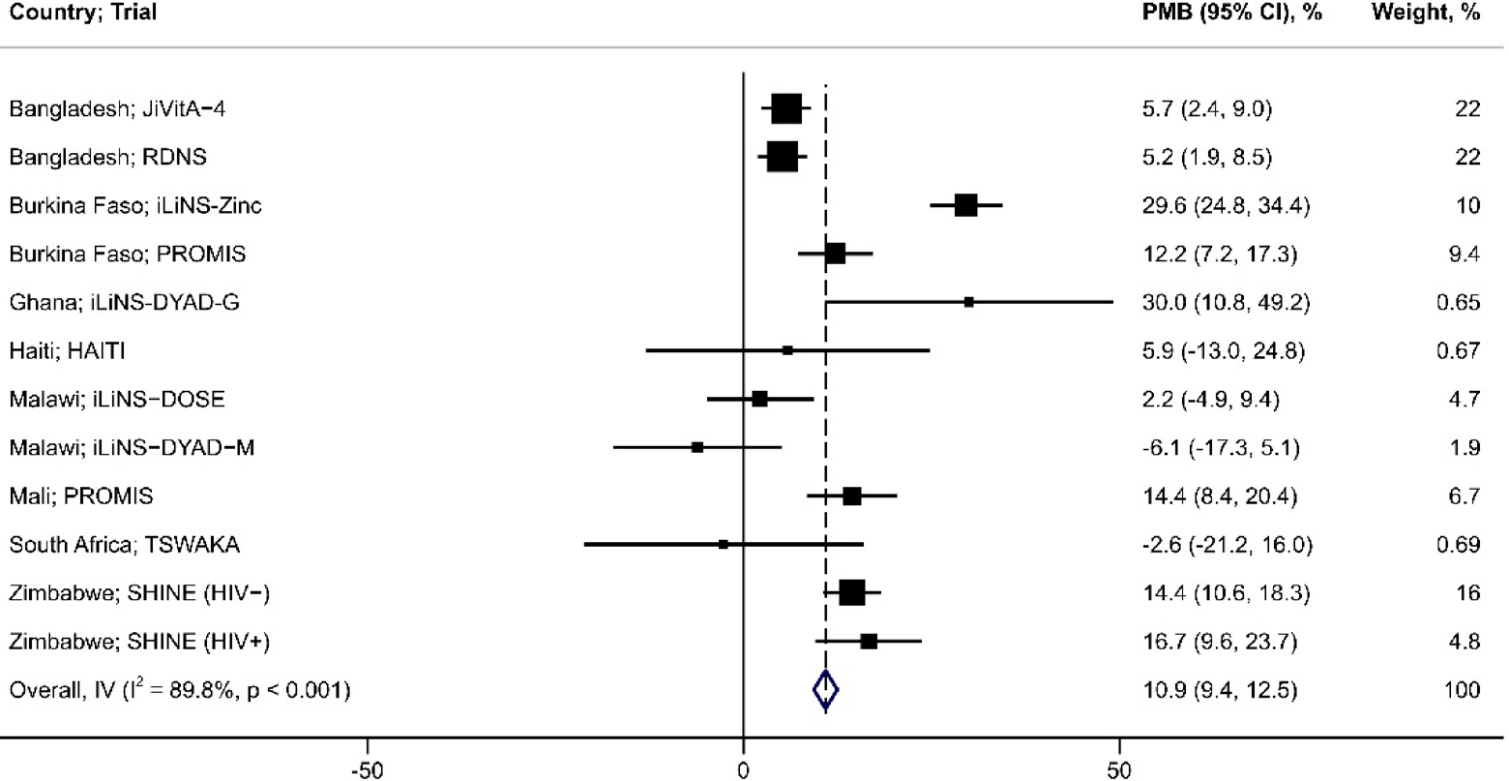
Effect of SQ-LNS expressed as the proportion of maximal benefit (PMB) (n = 19,768 participants, 12 comparisons). The results of individual trials were pooled to estimate an overall effect size using an inverse variance (IV) fixed-effects model. Blue diamond is the pooled effect with 95% CI; black boxes are centered at the point estimates for each comparison and the size of the box corresponds to its relative weight; horizontal lines extend to the lower and upper bounds of the 95% CI for the estimate for each comparison. CI, confidence interval; SQ-LNS, small-quantity lipid-based nutrient supplements.

Inferences and directions of the effects of SQ-LNS in each trial were generally consistent when expressed as the LAZ MD or height-age MD at end-line (Fig. 2). The largest LAZ MD was observed in the iLiNS-Zinc trial^28^, 0.3 (95% CI: 0.2, 0.4) (Fig. 3), which corresponded to a height-age MD of 20 days (95% CI: 12, 28) (Fig. 4). The smallest LAZ MD was observed in the TSWAKA trial^29^, − 0.13 (95% CI: − 0.34, 0.08) (Fig. 3), for which the height-age MD was -6 days (95% CI: − 18, 6) (Fig. 4). In the HAITI trial^30^, the end-line LAZ MD was 0.04 (95% CI: − 0.2, 0.3) (Fig. 3) whereas the height-age MD was 24 days (95% CI: 5, 43) (Fig. 4), a discordance that appeared as an outlier (Fig. 2). Across all trials, the correlation between the two effect estimates was moderate (Spearman rho = 0.74; *p* < 0.001) (Fig. 2a) but increased when the HAITI trial was excluded (Spearman rho = 0.94; *p* < 0.0001) (Fig. 2b). PMB ranged from − 6.1 to 30%, and the 25th, 50th, and 75th percentiles were 3.7%, 9.1%, and 16%, respectively (Fig. 5).

All three pooled effect measures indicated that SQ-LNS led to a significant average improvement in linear growth at trial endline (Table 2): mean LAZ was 0.15 higher in the SQ-LNS group compared to control (95% CI: 0.12, 0.17)(Fig. 3); SQ-LNS increased height-age by an average of 12 days compared to the control (95% CI: 9, 14)(Fig. 4); and, the PMB metric indicated that SQ-LNS achieves, on average, 11% of the growth potential (95% CI: 9.4, 12)(Fig. 5). The proportion of overall variance attributable to between-study heterogeneity (*I*^2^) was lowest when the effect was expressed as the LAZ MD and highest when expressed as the PMB (Table 2).

**Table 2:** Pooled estimates of the effect of SQ-LNS on child linear growth.

Outcomes	*n* Participants^1^ (Comparisons)	Effect estimate^2^ (95% CI)	*I*^2^, % (95% CI)
LAZ	36,970 (18)	0.15 (0.12, 0.17)	59 (21, 74)
HA, days	36,970 (18)	11.5 (9.4, 13.5)	60 (25, 75)
LAZ	19,768 (12)	0.13 (0.10, 0.16)	67 (28, 80)
HA, days	19,768 (12)	9.9 (7.5, 12.3)	69 (33, 81)
PMB, %	19,768 (12)	11 (9.4, 12)	90 (85, 93)

CI, confidence interval; HA, height-age; LAZ, length-for-age z-score; PMB, proportion of maximal benefit; SQ-LNS, small-quantity lipid-based nutrient supplements.

^1^Sample sizes correspond to number of participants and comparisons included in the meta-analysis.

^2^Mean difference (SQ-LNS group—control group) for all outcomes except the PMB.

Pooled effect estimates using random-effects models did not change the direction or interpretation of the effect of SQ-LNS on child linear growth (Supplementary Table S4). In a meta-analysis restricted to trials for which analyses were conducted using IPD, pooled estimates differed slightly from the corresponding values in the primary analysis, but the overall inferences and between-metric comparisons were unchanged when using the same approach as the primary analysis (Supplementary Figure S2) and when accounting for the cluster-randomized designs (Supplementary Figure S3). The meta-analytic inference was unchanged using mean raw length as the outcome measure (Supplementary Figure S4).

Simulations to quantify the effect of baseline imbalances in length on PMB showed that height-age discrepancies of 1 day may cause a bias of < 10 percentage points in the PMB in the null intervention effect scenario, but the effect is progressively more pronounced with greater discrepancies and/or when mean LAZ is closer to 0 (i.e., milder degree of prior faltering) (Supplementary Figure S5). The pattern of increasing bias in PMB with increasing baseline height-age differences was similar but slightly attenuated when simulating intervention effects that resulted in endline differences of 0.1 LAZ and 0.5 LAZ (Supplementary Figure S5).

## Discussion

In a meta-analysis of trials of infant SQ-LNS in LMICs, expression of endline between-group differences in height-age and estimation of the PMB provided alternative approaches to quantifying intervention effects compared to LAZ, a conventional metric which was the primary linear growth outcome in a previously published meta-analysis of SQ-LNS RCTs^8^. Whereas LAZ conveys differences in physical size expressed in z-score units that reflect each group’s deviation from a chronologically age-matched standard, height-age is an expression of each group’s stage of skeletal maturation (on the age scale) that is derived from raw length and is independent of the children’s chronological age. The PMB, derived from height-age, provides a method of benchmarking observed growth in an interval against a threshold based on established mechanisms of long bone growth and expected potential for recovery after faltering. However, whereas height-age and *growth delay* (chronological age minus height-age) have empirical and conceptual benefits in the description of population-average age-related linear growth patterns^16,17^, the advantages of height-age-derived metrics in RCT analysis remain uncertain.

We found a moderate-to-strong correlation between the effects of SQ-LNS reported as either the LAZ MD or height-age MD at trial end-line, and the two metrics generally led to the same statistical inferences, consistent with our expectations based on a prior pilot study of the methods to apply height-age-based metrics in RCT analysis^13^. However, differences between corresponding LAZ MD and height-age MD estimates may occur in a trial if the groups are imbalanced such that they have different length distributions at baseline. For example, in the HAITI trial^30^, the mean endline height-age of the intervention group (373 days) was significantly higher than the control group (354 days), whereas the corresponding mean LAZs were similar (− 0.70 vs − 0.74). In the absence of a treatment effect, this discrepancy could be explained by the between-group differences in chronological age at enrolment (~ 0.6 months) which we assumed to have persisted at endline; for a given LAZ, the chronologically older SQ-LNS group would have a higher raw mean length, and consequently a higher height-age. The inference from the height-age analysis of the HAITI trial was therefore similar to the comparison of raw lengths at endline (SQ-LNS: 74.3 cm vs control: 73.2 cm; MD: 1.06 (95% CI: 0.28, 1.84). Adjustment for baseline length (or LAZ) in a regression model, an approach often referred to as analysis of covariance (ANCOVA), yields unbiased estimates of the treatment effect expressed as a difference in raw length (or LAZ)^31,32^. However, since the conversion of length to height-age at the individual child level is practically challenging and conceptually problematic because most children are not expected to track along the WHO median curve^13^, the use of individual-level height-age data in an ANCOVA model may not be suitable for estimating a baseline-adjusted treatment effect on the height-age scale. The PMB is based on interval changes in height-age (∆HA) in each group and therefore offers a potential way to address baseline differences in length that are attributable to differences in chronological age (e.g., in the HAITI trial, the PMB was 6% and the confidence intervals overlapped the null). However, as discussed below, there may be limitations of the PMB in the context of other causes of baseline imbalances between trial groups.

Neither height-age MD nor the conventional metrics (LAZ MD, raw length MD) are benchmarked against the lower or upper bounds of plausible linear growth effects that could be achieved by an intervention in a defined age interval, and therefore the magnitudes of these effect estimates may be challenging to interpret and communicate in terms of tangible public health benefits. The perceived effectiveness of an intervention for improving linear growth on the LAZ scale is typically considered to be the extent to which mean LAZ in the treatment group approaches normal for chronological age (i.e., mean LAZ = 0 at trial end-line). For example, in the Kenya WASH-Benefits trial, the SQ-LNS intervention significantly increased endline mean LAZ compared to the control, but both groups’ mean LAZs remained low compared to the international standard (SQ-LNS: − 1.44; control: − 1.54)^9^. The study authors concluded that SQ-LNS interventions “can modestly reduce growth faltering but fall short of eliminating it,” implying that elimination corresponds to attainment of a mean LAZ of ~ 0^9^. Yet, even if an intervention fully remediated the entire range of complex factors believed to cause linear growth faltering^2^, it would be unlikely that a mean LAZ would reach 0 until pubertal growth is complete^16^. We developed the PMB metric in an effort to address this limitation by quantifying a short-term ∆HA in an intervention group in relation to two benchmarks: the children’s optimal growth potential in the same interval based on theoretical considerations (which defines the upper bound), and the maturation (∆HA) that occurred in the parallel control group over the same period (the lower bound). The PMB is calculated using height-age as there is no analogous method to define optimal interval growth of a population on the LAZ or raw length scales^16^. A PMB of 100% would indicate that an intervention essentially normalized growth although this is not a realistic target for a single intervention; yet a more plausible target threshold (i.e., < 100%) remains to be defined. Therefore, despite its appealing conceptual features, the interpretation and policy implications of the PMB remain challenging, and ultimately may depend on comparing PMBs across multiple interventions. A limitation of the present study is that we only considered a single type of intervention, and thus lack comparators for the PMB of 11% observed for SQ-LNS. However, it remains uncertain whether such comparisons of PMBs would lead to substantially different intervention rankings compared to what could be achieved by simply comparing the treatment effects on the LAZ or raw length scales.

A key consideration in the use of height-age, and by extension the PMB, is that the derivation of a height-age estimate is only valid for a population (or a representative sample of that population) for which mean length would be reasonably expected to adhere to the WHO median curve if there were no environmental constraints on growth (recognizing that fundamentally, the same assumption underlies the conventional use and interpretation of mean LAZ^3^). A further assumption is that any prior faltering (or recovery) was a whole-population phenomenon^33^, such that the mean length at any given age serves as a robust indicator of the suboptimal position of the population distribution relative to the WHO standard^17^. As noted earlier, height-age derivation using the WHO median curve is not intended to be applied to individual children, among whom we expect widely varying lengths at any given age in the absence of any delays in growth plate senescence (i.e., at the individual child level, length is assumed to be uncorrelated with skeletal maturation)^13^. Extending this idea further suggests that height-age should not be derived for a group of children who would be, on average, taller or shorter than the WHO standard even if growing optimally (e.g., a trial that enrolled only children with low birth weight). The SQ-LNS trials included in this SRMA had reasonably permissive inclusion criteria and therefore generally adhered to the assumption of whole-population representativeness. Yet, this does not necessarily imply that within each RCT, the randomly-selected groups all remained similarly representative of the underlying source population from which they were concurrently sampled. In fact, baseline between-group differences in length commonly arise by chance, despite similar chronological age distributions, leading to differences in baseline height-age that affect ∆HA and may therefore bias the PMB. When interpreting an estimate from a single RCT, our simulation study suggested it may be inadvisable to rely on the PMB when there is more than ~ 1 day difference in baseline height-age between treatment and control groups, particularly when it is not attributable to heterogeneity in the baseline chronological age distributions. Adherence to this recommendation would also mitigate regression to the mean (RTM), which can arise when comparing changes from baseline to endline between groups selected from the same underlying population, but which have different average baseline values^32^. Groups that differ in length at baseline due to chance are expected to display RTM by converging towards the overall population mean at endline, irrespective of any treatment effect^34^. However, since the direction of such biases in PMB estimation are expected to be randomly distributed across RCTs, they may be less consequential in the context of a meta-analysis.

In addition to chance imbalances in chronological age or average length, a third mechanism of between-group differences in baseline height-age occurs when the observation period begins *after* randomization. For example, in the iLiNS-DYAD trials in Ghana^35^ and Malawi^36^, the infant SQ-LNS intervention was essentially a continuation of a maternal SQ-LNS intervention in the same mother-infant pairs; and, in the Ghana trial, the maternal supplement had already caused the intervention group to have a greater length/LAZ than the control group by 6 months of age (the ‘baseline’ age for the infant intervention assessment)^35^. In this situation, we believe the PMB remains valid because the baseline height-age estimates are generated for groups that are no longer sub-samples of the same population but rather have distinct length distributions due to prior intervention effects. However, the ∆HA estimates remain subject to RTM artifacts.

Based on the *I*^2^ statistic, the impact of between-study heterogeneity was greater when the effect was expressed as the PMB compared to the LAZ MD or height-age MD, suggesting that pooling of the PMB should be approached more cautiously than the other outcome measures. The additional input variables required for PMB (i.e., baseline values, duration of the intervention period) may have amplified between-study variance, but this is difficult to discern from *I*^2^ since it is also affected by the scaling of the outcome measures^37^. Absolute measures of between-study variance (e.g., tau^2^) are expressed in the units of each outcome measure, so do not enable additional insights when comparing heterogeneity across outcomes. Meta-analyses of other health or nutritional interventions are needed to understand whether PMB has a consistently higher *I*^*2*^ than other outcome measures, which may suggest PMB is less suitable for pooling than other growth outcomes. Another limitation of our meta-analysis was that estimates generated by IPD analyses were combined with published study-level estimates. This is a practical approach to avoid selection biases when IPD is not available for all eligible studies, but can affect the heterogeneity assessment^38^. Also, our reliance on published reports reduced both the availability and precision of the data required for calculating height-age. Unmet data requirements for PMB substantially reduced the number of trials for which it could be calculated (n = 12) compared to height-age MD or LAZ MD at trial end-line (n = 18) because five trials were missing baseline length/LAZ^9,12,26,27,39^ and one trial was missing baseline data in the control group^40^. Although our overall inferences were unchanged when we restricted the analysis to the IPD trials, it may be preferable to conduct meta-analyses of height-age and PMB using IPD for all trials, which would also allow for analyses in subgroups defined by chronological age and sex.

Overall, the linear growth effect of an intervention such as SQ-LNS can be expressed in terms of height-age as an alternative to LAZ, if it is reasonable to assume that the WHO median growth curve is applicable to the source population of eligible trial participants. The PMB may further enhance the interpretability of effect estimates by quantifying the extent to which an intervention improves growth in relation to a biological threshold, but its practical value and validity remain uncertain, particularly under conditions of baseline imbalances in height-age arising from various mechanisms (e.g., chronological age discrepancies, random differences in length distributions, prior intervention effects). In meta-analyses, PMB may be affected by between-study heterogeneity to a greater extent than other measures, and may not be estimable when IPD are unavailable. Further research is needed to expand the application of height-age-based metrics to other interventions, to guide the interpretation of PMB for individual trials and in the context of meta-analyses, and to establish the utility and acceptability of PMB for comparisons of nutritional intervention effectiveness.

## Supplementary Information

Below is the link to the electronic supplementary material.

Supplementary Material 1

## Data Availability

Study-level data described in the manuscript that came from published sources can be found in the respective report, and the extracted dataset and code used in this manuscript is publicly available: https://github.com/kelly-watsonn/HA2.

## Abbreviations

CI: Confidence interval
HAZ: Height-for-age z-scores
IPD: Individual participant data
LAZ: Length-for-age z-score
LMICs: Low- and middle-income countries
MD: Mean difference
PMB: Proportion of maximal benefit
RCT: Randomized controlled trial
SQ-LNS: Small-quantity lipid-based nutrient supplements
SRMA: Systematic review and meta-analysis
WHO: World Health Organization
WHO-GS: WHO Growth Standards

## References

[1] Victora, C. G. et al. Revisiting maternal and child undernutrition in low-income and middle-income countries: Variable progress towards an unfinished agenda. *Lancet* **397**, 1388–1399. 10.1016/S0140-6736(21)00394-9 (2021). 33691094 10.1016/S0140-6736(21)00394-9 PMC7613170

[2] Black, R. E. et al. Maternal and child undernutrition and overweight in low-income and middle-income countries. *Lancet* **382**, 427–451. 10.1016/S0140-6736(13)60937-X (2013). 23746772 10.1016/S0140-6736(13)60937-X

[3] WHO child growth standards: length/height-for-age, weight-for-age, weight-for-length, weight-for-height and body mass index-for-age: methods and development. World Health Organization, 2006. Available at: https://www.who.int/tools/child-growth-standards.

[4] USAID. Stunting: Considerations for Use as an Indicator in Nutrition Projects., (USAID Advancing Nutrition, Arlington, VA, 2020).

[5] Frongillo, E. A., Leroy, J. L. & Lapping, K. Appropriate use of linear growth measures to assess impact of interventions on child development and catch-up growth. *Adv. Nutr.* **10**, 372–379. 10.1093/advances/nmy093 (2019). 30805630 10.1093/advances/nmy093 PMC6520037

[6] Vaivada, T. et al. What can work and how? An overview of evidence-based interventions and delivery strategies to support health and human development from before conception to 20 years. *Lancet* **399**, 1810–1829. 10.1016/s0140-6736(21)02725-2 (2022). 35489360 10.1016/S0140-6736(21)02725-2

[7] Dewey, K. G. et al. Characteristics that modify the effect of small-quantity lipid-based nutrient supplementation on child growth: An individual participant data meta-analysis of randomized controlled trials. *Am. J. Clin. Nutr.* **114**, 15s–42s. 10.1093/ajcn/nqab278 (2021). 34590672 10.1093/ajcn/nqab278 PMC8560308

[8] Dewey, K. G. et al. Lipid-based nutrient supplementation in the first 1000 d improves child growth in Bangladesh: A cluster-randomized effectiveness trial1, 2, 3. *Am. J. Clin. Nutr.* **105**, 944–957. 10.3945/ajcn.116.147942 (2017). 28275125 10.3945/ajcn.116.147942

[9] Null, C. et al. Effects of water quality, sanitation, handwashing, and nutritional interventions on diarrhoea and child growth in rural Kenya: A cluster-randomised controlled trial. *Lancet Glob. Health* **6**, e316–e329. 10.1016/s2214-109x(18)30005-6 (2018). 29396219 10.1016/S2214-109X(18)30005-6 PMC5809717

[10] Christian, P. et al. Effect of fortified complementary food supplementation on child growth in rural Bangladesh: A cluster-randomized trial. *Int. J. Epidemiol.* **44**, 1862–1876. 10.1093/ije/dyv155 (2015). 26275453 10.1093/ije/dyv155 PMC4689999

[11] Prendergast, A. J. et al. Independent and combined effects of improved water, sanitation, and hygiene, and improved complementary feeding, on stunting and anaemia among HIV-exposed children in rural Zimbabwe: A cluster-randomised controlled trial. *Lancet Child Adolesc. Health* **3**, 77–90. 10.1016/s2352-4642(18)30340-7 (2019). 30573417 10.1016/S2352-4642(18)30340-7 PMC6472652

[12] Luby, S. P. et al. Effects of water quality, sanitation, handwashing, and nutritional interventions on diarrhoea and child growth in rural Bangladesh: A cluster randomised controlled trial. *Lancet Glob. Health* **6**, e302–e315. 10.1016/S2214-109X(17)30490-4 (2018). 29396217 10.1016/S2214-109X(17)30490-4 PMC5809718

[13] Watson, K. M. et al. Height-age as an alternative to height-for-age z-scores to assess the effect of interventions on child linear growth in low- and middle-income countries. *Curr. Dev. Nutr.* **8**, 104495. 10.1016/j.cdnut.2024.104495 (2024). 39649476 10.1016/j.cdnut.2024.104495 PMC11621485

[14] Lui, J. C., Nilsson, O. & Baron, J. Growth plate senescence and catch-up growth. *Endocr. Dev.* **21**, 23–29. 10.1159/000328117 (2011). 21865751 10.1159/000328117 PMC3420820

[15] Gat-Yablonski, G. & Phillip, M. Nutritionally-induced catch-up growth. *Nutrients* **7**, 517 (2015). 25594438 10.3390/nu7010517 PMC4303852

[16] Mansukoski, L. et al. Growth delay: An alternative measure of population health based on child height distributions. *Ann. Hum. Biol.* **49**, 100–108. 10.1080/03014460.2022.2091794 (2022). 35736806 10.1080/03014460.2022.2091794

[17] Aimone, A. M. et al. Complementary and alternative metrics for tracking population-level trends in child linear growth. *PLOS Glob. Public Health* **3**, e0001766. 10.1371/journal.pgph.0001766 (2023). 37068059 10.1371/journal.pgph.0001766 PMC10109512

[18] Das, J. K. et al. Preventive lipid-based nutrient supplements given with complementary foods to infants and young children 6 to 23 months of age for health, nutrition, and developmental outcomes. *Cochrane Database Syst. Rev.* **5**, Cd012611. 10.1002/14651858.CD012611.pub3 (2019). 31046132 10.1002/14651858.CD012611.pub3 PMC6497129

[19] Higgins, J. P. T. & Green, S. Cochrane Handbook for Systematic Reviews of Interventions Version 5.1.0 (updated March 2011). The Cochrane Collaboration, 2011. Available from training.cochrane.org/handbook/archive/v5.1/.

[20] WHO. *Child growth standards - The WHO Anthro Software Packages and Macros STATA macro igrowup_update. Available at: *https://www.who.int/tools/child-growth-standards/software.

[21] Watson, K. M. *HA1*, https://github.com/kelly-watsonn/HA1 (2024).

[22] StataCorp. 2021. Stata Statistical Software: Release 17. College Station, TX: StataCorp LLC.

[23] Higgins, J. P. *et al.* Cochrane Handbook for Systematic Reviews of Interventions Version 6.5 (updated August 2024). Available from https://training.cochrane.org/handbook. *Cochrane Community* (2024).

[24] Anderson, C., Xiao, L. & Checkley, W. Using data from multiple studies to develop a child growth correlation matrix. *Stat. Med.* **38**, 3540–3554. 10.1002/sim.7696 (2019). 29700850 10.1002/sim.7696 PMC6767589

[25] Humphrey, J. H. et al. Independent and combined effects of improved water, sanitation, and hygiene, and improved complementary feeding, on child stunting and anaemia in rural Zimbabwe: A cluster-randomised trial. *Lancet Glob. Health* **7**, e132–e147. 10.1016/S2214-109X(18)30374-7 (2019). 30554749 10.1016/S2214-109X(18)30374-7 PMC6293965

[26] Becquey, E. et al. Impact on child acute malnutrition of integrating a preventive nutrition package into facility-based screening for acute malnutrition during well-baby consultation: A cluster-randomized controlled trial in Burkina Faso. *PLoS Med.* **16**, e1002877. 10.1371/journal.pmed.1002877 (2019). 31454347 10.1371/journal.pmed.1002877 PMC6711504

[27] Huybregts, L. et al. Impact on child acute malnutrition of integrating small-quantity lipid-based nutrient supplements into community-level screening for acute malnutrition: A cluster-randomized controlled trial in Mali. *PLoS Med.* **16**, e1002892. 10.1371/journal.pmed.1002892 (2019). 31454356 10.1371/journal.pmed.1002892 PMC6711497

[28] Hess, S. Y. et al. Small-quantity lipid-based nutrient supplements, regardless of their zinc content, increase growth and reduce the prevalence of stunting and wasting in young burkinabe children: A cluster-randomized trial. *PLoS ONE* **10**, e0122242. 10.1371/journal.pone.0122242 (2015). 25816354 10.1371/journal.pone.0122242 PMC4376671

[29] Smuts, C. M. et al. Effect of small-quantity lipid-based nutrient supplements on growth, psychomotor development, iron status, and morbidity among 6- to 12-mo-old infants in South Africa: A randomized controlled trial. *Am. J. Clin. Nutr.* **109**, 55–68. 10.1093/ajcn/nqy282 (2019). 30649163 10.1093/ajcn/nqy282 PMC6358035

[30] Iannotti, L. L. et al. Linear growth increased in young children in an urban slum of Haiti: A randomized controlled trial of a lipid-based nutrient supplement. *Am. J. Clin. Nutr.* **99**, 198–208. 10.3945/ajcn.113.063883 (2014). 24225356 10.3945/ajcn.113.063883 PMC3862455

[31] Zhang, S. et al. Empirical comparison of four baseline covariate adjustment methods in analysis of continuous outcomes in randomized controlled trials. *Clin. Epidemiol.* **6**, 227–235. 10.2147/CLEP.S56554 (2014). 25053894 10.2147/CLEP.S56554 PMC4105274

[32] Egbewale, B. E., Lewis, M. & Sim, J. Bias, precision and statistical power of analysis of covariance in the analysis of randomized trials with baseline imbalance: A simulation study. *BMC Med. Res. Methodol.* **14**, 49. 10.1186/1471-2288-14-49 (2014). 24712304 10.1186/1471-2288-14-49 PMC3986434

[33] Roth, D. E. et al. Early childhood linear growth faltering in low-income and middle-income countries as a whole-population condition: analysis of 179 Demographic and Health Surveys from 64 countries (1993–2015). *Lancet Glob. Health* **5**, e1249–e1257. 10.1016/S2214-109X(17)30418-7 (2017). 29132614 10.1016/S2214-109X(17)30418-7 PMC5695758

[34] Linden, A. Assessing regression to the mean effects in health care initiatives. *BMC Med. Res. Methodol.* **13**, 119. 10.1186/1471-2288-13-119 (2013). 24073634 10.1186/1471-2288-13-119 PMC3849564

[35] Adu-Afarwuah, S. et al. Small-quantity, lipid-based nutrient supplements provided to women during pregnancy and 6 mo postpartum and to their infants from 6 mo of age increase the mean attained length of 18-mo-old children in semi-urban Ghana: A randomized controlled trial. *Am. J. Clin. Nutr.* **104**, 797–808. 10.3945/ajcn.116.134692 (2016). 27534634 10.3945/ajcn.116.134692 PMC4997301

[36] Ashorn, P. et al. Supplementation of maternal diets during pregnancy and for 6 months postpartum and infant diets thereafter with small-quantity lipid-based nutrient supplements does not promote child growth by 18 months of age in rural Malawi: A randomized controlled trial. *J. Nutr.* **145**, 1345–1353. 10.3945/jn.114.207225 (2015). 25926413 10.3945/jn.114.207225

[37] Higgins, J. P., Thompson, S. G., Deeks, J. J. & Altman, D. G. Measuring inconsistency in meta-analyses. *BMJ* **327**, 557–560. 10.1136/bmj.327.7414.557 (2003). 12958120 10.1136/bmj.327.7414.557 PMC192859

[38] Veroniki, A. A., Seitidis, G., Tsivgoulis, G., Katsanos, A. H. & Mavridis, D. An Introduction to individual participant data meta-analysis. *Neurology* **100**, 1102–1110. 10.1212/WNL.0000000000207078 (2023). 36797070 10.1212/WNL.0000000000207078 PMC10256124

[39] Galasso, E., Weber, A. M., Stewart, C. P., Ratsifandrihamanana, L. & Fernald, L. C. H. Effects of nutritional supplementation and home visiting on growth and development in young children in Madagascar: A cluster-randomised controlled trial. *Lancet Glob. Health* **7**, e1257–e1268. 10.1016/S2214-109X(19)30317-1 (2019). 31402006 10.1016/S2214-109X(19)30317-1

[40] Adu-Afarwuah, S. et al. Randomized comparison of 3 types of micronutrient supplements for home fortification of complementary foods in Ghana: Effects on growth and motor development. *Am. J. Clin. Nutr.* **86**, 412–420. 10.1093/ajcn/86.2.412 (2007). 17684213 10.1093/ajcn/86.2.412

