# Supplementary material for: Quantifying child growth effects using height-age instead of height-for-age z-scores in a meta-analysis of small-quantity lipid-based nutrient supplement trials
Source: Sci Rep. 2025 Oct 22;15:36853. doi: 10.1038/s41598-025-20664-9 (PMC12546833; doi:10.1038/s41598-025-20664-9)
Supplement: Supplementary file 1 — Supplementary Material 1 [file 41598_2025_20664_MOESM1_ESM.pdf]

Supplementary Appendix

Height-age instead of height-for-age z-scores to quantify linear growth effects of nutritional interventions in randomized controlled trials

Kelly M Watson, Alison SB Dasiewicz, Diego G Bassani, Chun-Yuan Chen,

Huma Qamar, Daniel E Roth

Supplementary Table S1. Study information extracted from published SQ-LNS trial reports.

Study information	First author's last name<br>Publication year<br>Trial name<br>Title of report of trial
Methods	Design (e.g., multi-arm, randomized controlled trial)<br>Unit of randomization (individual or cluster)<br>Type of follow-up (longitudinal or cross-sectional)<br>WHO-GS used to generate z-scores (Y/N)
Participants	Geographical location (country)<br>Inclusion criteria<br>Exclusion criteria
Interventions	Relevant intervention(s) (all groups combined into the SQ-LNS group)<br>Other intervention groups (not included in the comparison for this study)<br>Relevant control group(s) (all groups combined into the control group)<br>Duration of intervention period (period of SQ-LNS supplementation)<br>Average age at start of SQ-LNS supplementation<br>Primary end-line assessed in the trial
Outcomes	Primary outcome(s)<br>Secondary outcome(s)<br>Timing of linear growth outcome assessments
Results	Effect of SQ-LNS on linear growth
Other	Additional comments

Supplementary Table S2. Data extracted from published SQ-LNS trial reports.

Variable	Definition	Form Instructions
Study ID		First author's last name and publication year
Group name		Complete a separate form for all relevant intervention and control groups
Time	Time of assessment	Input "0" for baseline or "1" for primary end-line
Duration of intervention		Enter in the format: number (unit)
Sample size of group		If there are multiple sample sizes reported at a specific follow-up time point, use the smallest reported sample size
G_N	Number of females in group	
G_%	Percentage of females in group	
G_prop	Proportion of females in group	
B_N	Number of males in group	
B_%	Percentage of males in group	
B_prop	Proportion of males in group	
Age	Average (mean or median) age at time of assessment	Only extract as raw value (i.e., if not reported at end-line, leave blank)
Unit_age	Unit of time which the average age is reported in	Input: "years", "months", or "days"
SD_age	Standard deviation of age	
LB_age	Lower bound (LB) of average age 95% CI	Specify confidence level other than 95%, format as: LB (level)
UB_age	Upper bound (UB) of average age 95% CI	Specify confidence level other than 95%, format as: UB (level)
Age_range	Range of ages included at the time of assessment	
Length	Average (mean or median) length	
Unit_length	Unit that length is reported in	Input: "cm", "m"
SD_length	Standard deviation of length	
LB_length	Lower bound (LB) of average length 95% CI	Specify confidence level other than 95%, format as: LB (level)
UB_length	Upper bound (UB) of average length 95% CI	Specify confidence level other than 95%, format as: UB (level)
Height	Average (mean or median) height	
Unit_height	Unit average height reported in	Input: "cm", "m"
SD_height	Standard deviation of height	

Variable	Definition	Form Instructions
LB_height	Lower bound (LB) of average height 95% CI	Specify confidence level other than 95%, format as: LB (level)
UB_height	Upper bound (UB) of average height 95% CI	Specify confidence level other than 95%, format as: UB (level)
LAZ	Average (mean or median) LAZ	
SD_LAZ	Standard deviation of LAZ	
LB_LAZ	Lower bound (LB) of average LAZ 95% CI	Specify confidence level other than 95%, format as: LB (level)
UB_LAZ	Upper bound (UB) of average LAZ 95% CI	Specify confidence level other than 95%, format as: UB (level)
HAZ	Average (mean or median) HAZ	
SD_HAZ	Standard deviation of HAZ	
LB_HAZ	Lower bound (LB) of average HAZ 95% CI	Specify confidence level other than 95%, format as: LB (level)
UB_HAZ	Upper bound (UB) of average HAZ 95% CI	Specify confidence level other than 95%, format as: UB (level)

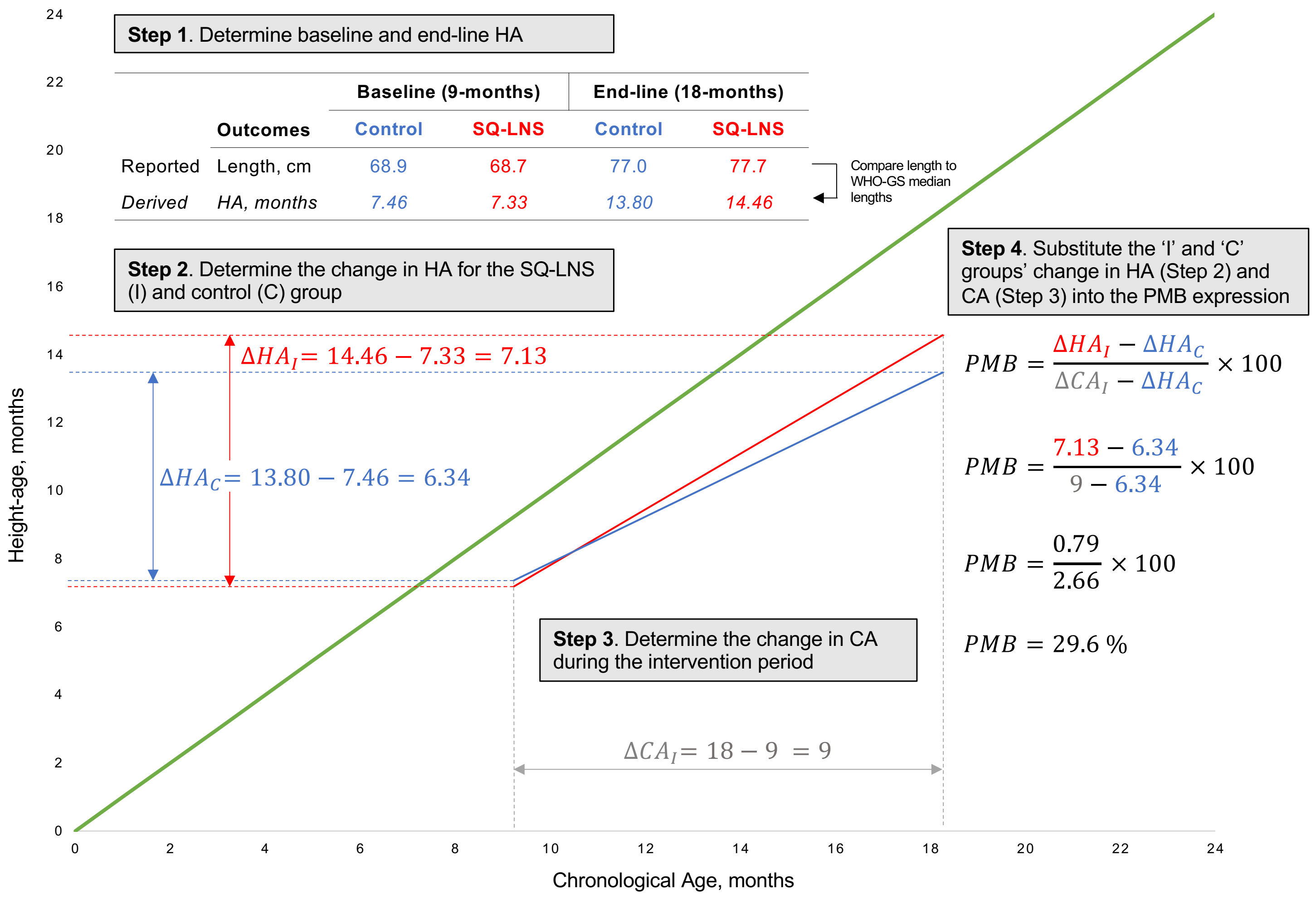

Supplementary Figure S1. Calculation of the proportion of maximal benefit (PMB). Subscript 'I' represents the intervention (SQ-LNS) group, subscript 'C' represents the control group. Changes (Δs) are calculated from baseline to end-line. Steps shown for illustration purposes only. PMB show was calculated using unrounded input numbers. Abbreviations: CA, chronological age; HA, height-age; SQ-LNS, small-quantity lipid-based nutrient supplements; WHO-GS, World Health Organization Growth Standards. Data source: Hess SY, Abbeddou S, Jimenez EY, Somé JW, Vosti SA, Ouédraogo ZP, et al. Small-Quantity Lipid-Based Nutrient Supplements, Regardless of Their Zinc Content, Increase Growth and Reduce the Prevalence of Stunting and Wasting in Young Burkinabe Children: A Cluster-Randomized Trial. PLOS ONE. 2015; 10(3):e0122242.

Supplementary Table S3. Data availability for calculating height-age among included studies.

	Baseline			Endline	Publicly available IPD?	Contacted authors?[1]	Approach used to calculate HA
**Country; trial**	**LAZ/len**	**Age**	**Sex %**	**LAZ/len**			
Bangladesh; JiVitA	Both*	Yes*	Yes*, [2]	Both*	No	No	Mean-length
Bangladesh; RDNS	No	No	No	LAZ*	No	Yes[3]	Mean-LAZ
Bangladesh; WASH-B	NA			LAZ*	Yes[4]	No	Mean-length
Burkina Faso; iLiNS-ZINC	Both*	Yes*	Yes*	Both*	No	No	Mean-length
Burkina Faso; PROMIS	No	Yes*	Yes*	No	Yes[4]	No	Mean-length
Ghana; GHANA	NA			LAZ*	No	No	Mean-LAZ
Ghana; iLiNS-DYAD-G[5]	LAZ*	No	No	Both*	No	No	Both[6]
Haiti; HAITI	LAZ*	Yes*, [7]	Yes*	LAZ*	No	Yes[8]	Mean-length
Kenya; WASH-B	NA			LAZ*	Yes[4]	No	Mean-length
Madagascar; MAHAY	NA			LAZ*	No	No	Mean-LAZ
Malawi; iLiNS-DYAD-M	LAZ*	No	No	Both*	No	No	Both[6]
Malawi; iLiNS-DOSE	Both*	Yes*	Yes*	Both*	No	No	Mean-length
Mali; PROMIS	No	Yes*	Yes*	No	Yes[4]	No	Mean-length
South Africa; TSWAKA	Both*	Yes*	Yes*	Both*	No	No	Mean-length
Zimbabwe; SHINE (HIV-)	No	No	Yes*	LAZ*	No	Yes[9]	Mean-LAZ
Zimbabwe; SHINE (HIV+)	No	No	Yes*	LAZ*	No	Yes[9]	Mean-LAZ

Abbreviations: CI, confidence interval; HA, height-age; IPD, individual participant data; LAZ, length-for-age z-score; len, raw length; SD, standard deviation.
* Reported by group.
[1] Only contacted authors if length/LAZ data was missing. If the average age of children was not reported, assumed the average age was equal to the time of the scheduled measurement. If the ratio of males to females was not reported, we assumed it was equal; if not reported at the end-line, we assumed it was the same as baseline.
[2] Male percentage in Table 2 of the trial report was incorrect – extracted the number of males only and calculated the correct percentage.
[3] Authors provided 6-month LAZ data (mean, standard errors, and 95% CI) and age (mean and SDs), by group.
[4] Mean length (SD), LAZ (SD), age (SD), and sex ratio, by group, were calculated using public IPD for our analyses.

[5] Protocol violation – extracted data corresponding to supplements received.
[6] Mean-length used to determine end-line height-age; mean-LAZ used to determine baseline height-age.
[7] Groups had statistically significant different chronological ages at baseline: Control: 7.2 (SD 1.7) months; 6-mo LNS: 7.8 (SD 1.7) months.
[8] Authors provided baseline and end-line length (SD), by group.
[9] Authors provided 6-month LAZ (mean, SEs, and 95% CI) and age (mean and SDs) data, by group, disaggregated by HIV exposure.

Supplementary Table S4. Meta-analysis using random-effects versus fixed-effects models to pool measures of effect of SQ-LNS on linear growth.

Outcomes	*n* Participants (Comparisons)	Effect Estimate (95% CI)[1], IV Fixed	Effect Estimate (95% CI)[1], IV Random
LAZ	36970 (18)	0.15 (0.12, 0.17)	0.14 (0.10, 0.18)
Height-age, days	36970 (18)	11.5 (9.4, 13.5)	11.7 (8.2, 15.2)
LAZ	19768 (12)	0.13 (0.10, 0.16)	0.13 (0.07, 0.19)
Height-age, days	19768 (12)	9.9 (7.5, 12.3)	10.3 (5.7, 15.0)
PMB, %	19768 (12)	11 (9.4, 12)	11 (5.5, 16)

Abbreviations: CI, confidence interval; IV, inverse variance; LAZ, length-for-age z-score; PMB, proportion of maximal benefit; SQ-LNS, small-quantity lipid-based nutrient supplements.
[1] Mean difference (SQ-LNS group – control group) for all outcomes except the PMB.

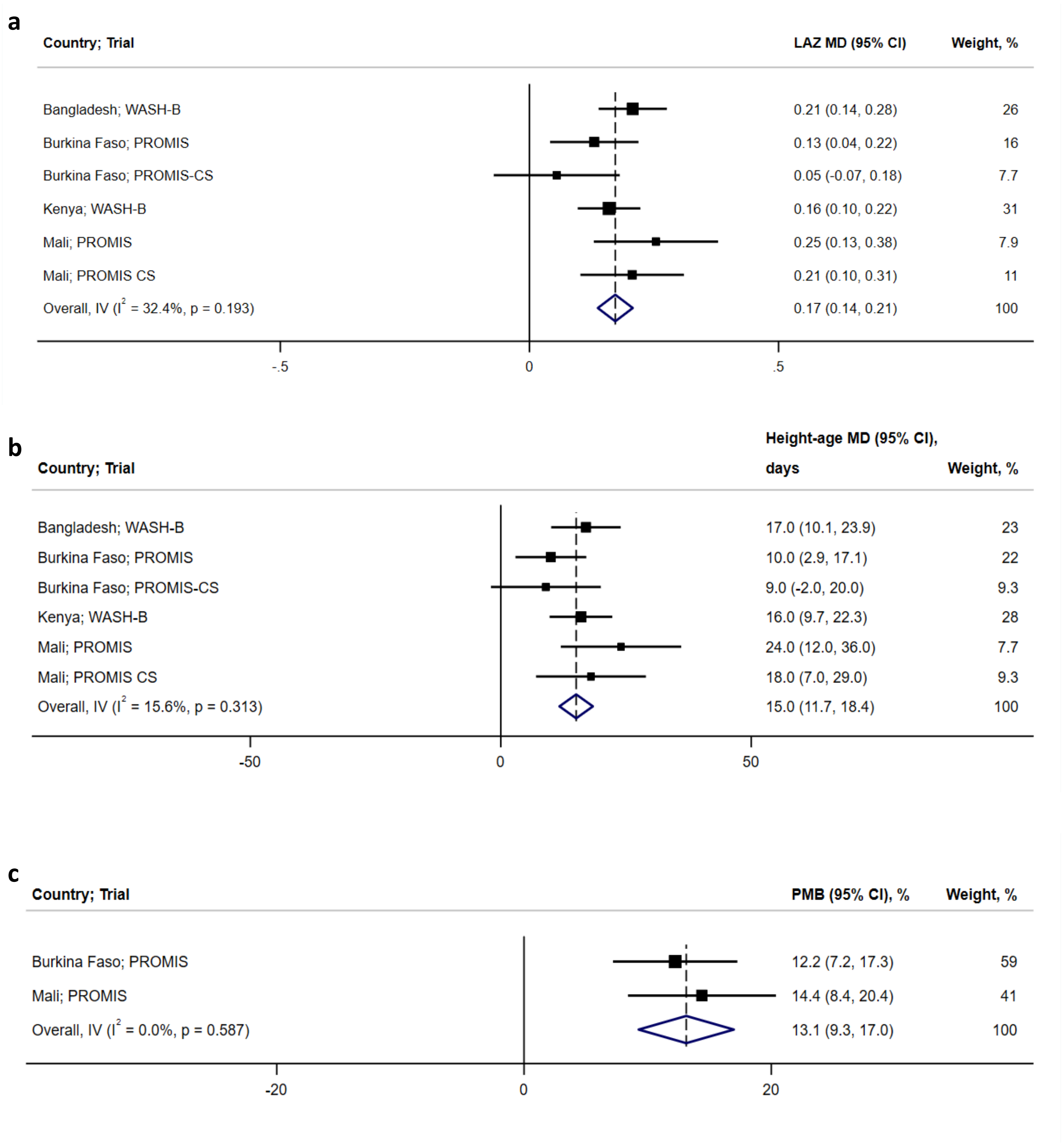

Supplementary Figure S2. Effect of SQ-LNS on linear growth expressed as the LAZ mean difference (SQ-LNS vs control), height-age mean difference at trial endline (SQ-LNS vs control) [n=16840, 6 comparisons] and proportion of maximal benefit [n=2731, 2 comparisons] among trials with available individual participant data. The results of individual trials were pooled to estimate an overall effect size using an inverse variance (IV) fixed-effects model. Symbols: Blue diamond is the pooled effect with 95% CI; Black boxes are centered at the point estimates for each comparison and the size of the box corresponds to its relative weight; horizontal lines extend to the lower and upper bounds of the 95% CI for the estimate for each comparison. Abbreviations: CI, confidence interval; LAZ, length-for-age z-score; MD, mean difference; PMB, proportion of maximal benefit; SQ-LNS, small-quantity lipid-based nutrient supplements.

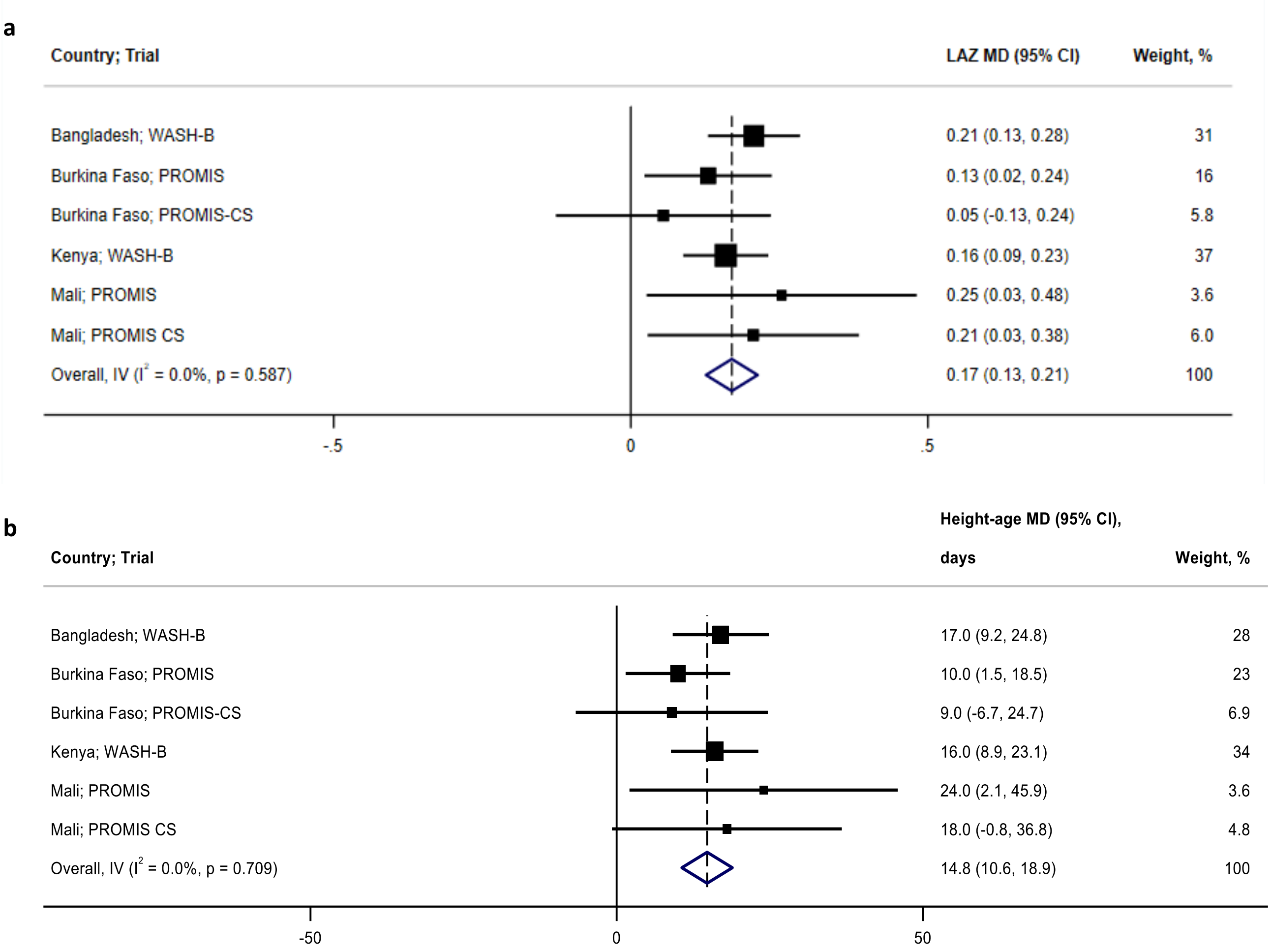

Supplementary Figure S3. Effect of SQ-LNS on linear growth expressed as the LAZ mean difference (SQ-LNS vs control), height-age mean difference at trial endline (SQ-LNS vs control) [n= 11323, 6 comparisons] among trials with available individual participant data, accounting for the cluster-randomized trial design. The results of individual trials were pooled to estimate an overall effect size using an inverse variance (IV) fixed-effects model. Symbols: Blue diamond is the pooled effect with 95% CI; Black boxes are centered at the point estimates for each comparison and the size of the box corresponds to its relative weight; horizontal lines extend to the lower and upper bounds of the 95% CI for the estimate for each comparison. Abbreviations: CI, confidence interval; LAZ, length-for-age z-score; MD, mean difference; SQ-LNS, small-quantity lipid-based nutrient supplements.

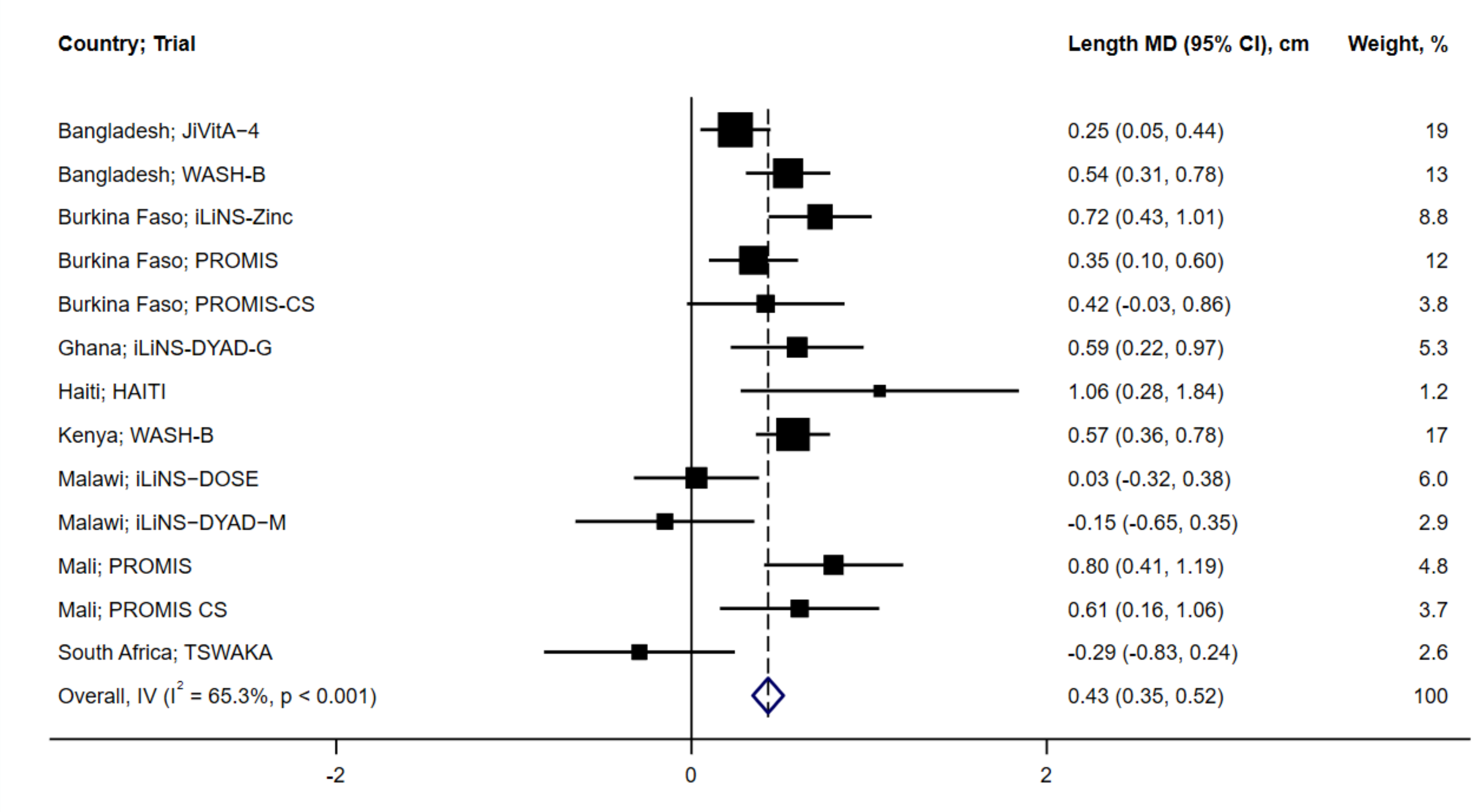

Supplementary Figure S4. Effect of SQ-LNS on linear growth expressed as the difference in endline mean raw length [n=27056, 13 comparisons]. The results of individual trials were pooled to estimate an overall effect size using an inverse variance (IV) fixed-effects model. Symbols: Blue diamond is the pooled effect with 95% CI; Black boxes are centered at the point estimates for each comparison and the size of the box corresponds to its relative weight; horizontal lines extend to the lower and upper bounds of the 95% CI for the estimate for each comparison. Abbreviations: CI, confidence interval; MD, mean difference; SQ-LNS, small-quantity lipid-based nutrient supplements.

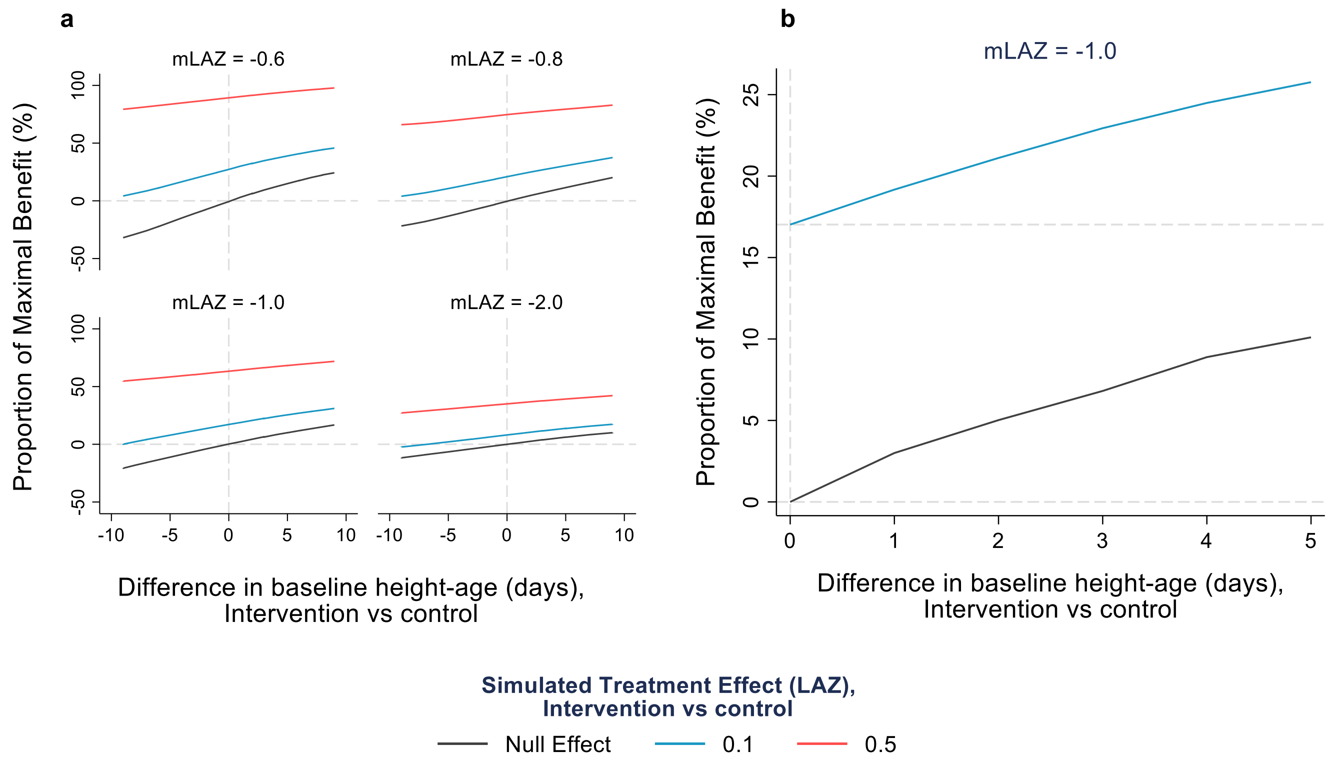

Supplementary Figure S5. Proportion of maximal benefit (PMB) at varying levels of between-group baseline height-age differences, by overall population mean LAZ (mLAZ), in simulated randomized controlled trials of hypothetical interventions. Simulations were of 10,000 participants in clinical trials divided into a single intervention arm and a control arm. The simulation was repeated at varying levels of baseline differences in height-age and prior linear growth faltering. In each scenario, we induced the same absolute intervention effect on the LAZ scale to visualize the extent of bias introduced by varying levels of baseline height-age differences in different populations. Panel **(a)** shows the PMB as a function of baseline differences when there is no difference (null effect), a 0.1-unit increase, or a 0.5-unit increase in LAZ in the intervention group versus control at endline. The vertical dashed line intersects each curve at the estimated PMB when there is no between-group baseline difference in height-age. Panel **(b)** represents the same simulation but zoomed in to show baseline height-age differences ranging from 0 to 5 days in the scenarios involving a no-difference (null effect) and 0.1-unit increase in LAZ in the intervention group versus control in populations with mean LAZ of -1. The dashed horizontal lines represent the reference PMB when there are no baseline differences in height-age.
